# The Spectrum of Non-dermatophyte Onychomycosis With Special Reference to Syncephalastrum as an Emerging Pathogen

**DOI:** 10.7759/cureus.90632

**Published:** 2025-08-20

**Authors:** Uneza Husain, Nidhi Negi, Yogita Rawat, Shekhar Pal, Ruhi Hassan

**Affiliations:** 1 Department of Microbiology, Jawaharlal Institute of Postgraduate Medical Education and Research, Puducherry, IND; 2 Department of Microbiology, Government Doon Medical College, Dehradun, IND

**Keywords:** fungi, fungus, non-dermatophyte, onychomycosis, syncephalastrum

## Abstract

Clinical and epidemiological research has shown that non-dermatophyte molds and yeasts account for 20-30% of onychomycosis cases. Hence, in the present study, a retrospective evaluation of suspected cases of onychomycosis over one year and one month, from January 2022 to January 2023, was conducted. Out of 44 cases, non-dermatophyte fungi were isolated in eight (18%) nail specimens on culture. We detected four isolates of *Aspergillus *spp. (including two isolates each of *Aspergillus flavus* and* Aspergillus niger*), two isolates of *Candida *spp.,oneisolate of * Fusarium *spp., and one isolate of *Syncephalastrum *spp. by culture. We also briefly described *the Syncephalastrum*-related review of literature, as this case is one of the rarest in India, although a few cases have been reported worldwide.

## Introduction

Onychomycosis (fungal infection of the nails) is caused by dermatophytes, non-dermatophyte molds, and yeasts [1,2]. Based on recent epidemiological studies, the disease is known to have an estimated prevalence of 5.5% worldwide and 2-14% in the United States [3,4]. Traditionally, dermatophytes, predominantly *Trichophyton* species, have been implicated as the commonest cause of onychomycosis, but contrary to this, in recent years, there has been an increase in the reported cases of non-dermatophytes as etiological agents of onychomycosis [2]. The possible cause might be an improvement in diagnostics and ongoing extensive research in this dynamic era of updated technology. Although microscopy and culture have traditionally been the standard, newer methods like polymerase chain reaction (PCR) offer greater sensitivity and specificity [2]. Due to the high recurrence rate, long-term maintenance therapy with topical antifungals is used in most cases [3]. The present study aims to evaluate the suspected cases of onychomycosis caused by non-dermatophyte fungi. There is a scarcity of studies on onychomycosis caused by non-dermatophytes in India, particularly in the region of Uttarakhand. Hence, the present study will be a useful addition to the existing literature.

Objectives

This study aimed (a) to study the spectrum of non-dermatophyte onychomycosis cases in patients presenting at Government Doon Medical College, Dehradun, (b) to evaluate direct microscopic examination (potassium hydroxide) findings that helped in rapid presumptive fungal identification in nail specimens, (c) to analyze the final identification results by lactophenol cotton blue (LPCB) mount based on growth from culture, and (d) to perform a brief literature review in the context of* Syncephalastrum* as an emerging pathogen.

## Materials and methods

Study design

This study is a retrospective analysis of data collected from suspected cases of onychomycosis. The data were sourced from medical records of patients who underwent nail specimen collection and were tested in the microbiology laboratory for the presence of fungi during one year and one month from January 2022 to January 2023. The data included patient demographics and laboratory results. Only cases with complete data records were included in the analysis.

Study site

The study was conducted at Government Doon Medical College in Dehradun, a leading medical institution in northern India. The college serves a diverse patient population from Dehradun and the surrounding regions, which makes it an ideal setting for conducting this study on suspected cases of onychomycosis.

Study participants

A total of 44 patients with suspected onychomycosis were evaluated in the study, including the outpatient department (OPD) and/or indoor patients presenting at Government Doon Medical College, Dehradun. 

Sampling

Nail specimens were collected in a sterile petri dish with the help of a sterile scalpel blade. All sampling procedures were conducted in a controlled, sterile environment to minimize the risk of introducing external contaminants into the samples. Each specimen was clearly labeled with the patient's identification information and collection date to maintain proper record-keeping and ensure accurate tracking throughout the analysis process.

Procedure

Nail specimens were analyzed, and the results were categorized as positive or negative for non-dermatophyte fungi. We performed direct microscopy (20-40% potassium hydroxide (KOH) was used) and mycological culture using tubes containing Sabouraud dextrose agar (SDA) media (with and without chloramphenicol). The tubes were kept at 25°C and 37°C for incubation. We performed an LPCB mount from the growth and observed microscopically for the presence of conidiation/sporulation/hyphae and other microscopic features of different fungi. Additionally, we performed a slide culture for better identification of morphological characteristics.

Data analysis

In this study, we transferred all pertinent data from the laboratory information system into a Microsoft Excel spreadsheet (Microsoft 365 version 2507, Microsoft Corporation, Redmond, Washington, United States). We calculated the percentage of cases that tested positive for non-dermatophyte fungi in the nail specimens. This involved determining the total number of positive cases and dividing it by the total number of cases analyzed and then multiplying by 100 to obtain the percentage positivity. All cases of non-dermatophyte onychomycosis (all age groups) detected by microscopy and/or only culture were included in the study. Onychomycosis cases caused by dermatophytes were excluded from the study.

Statistical analysis

The optimal sample size was found to be 40 using Yamane's statistical formula [5]: n=N/1+N(e)2. Here, n is the required sample size from the population under study, N is the whole population that is under study, and e is the level of precision. Descriptive statistics were employed, with categorical data presented as frequencies or proportions.

Ethics

Ethics clearance was obtained from the Institutional Ethics Committee of Government Doon Medical College (approval number: GDMC/IEC/2023/45) before the commencement of this study.

## Results

Out of 44 suspected cases of onychomycosis, non-dermatophytes were isolated in eight (18%) nail specimens on culture. We detected four isolates of *Aspergillus* spp., including *Aspergillus flavus* (2) and *Aspergillus niger* (2), two isolates of *Candida *spp., one isolate of *Fusarium *spp., and one isolate of *Syncephalastrum* spp. in nail specimens by culture (Table 1).

**Table 1:** Data of non-dermatophyte fungi isolated from nail specimens between January 2022 and January 2023 KOH: potassium hydroxide

S. no.	Age	Sex	Organism isolated in culture	KOH findings
1	21	Male	Aspergillus niger	Thin, hyaline, septate, branched fungal hyphae seen
2	20	Female	Aspergillus flavus	Thin, hyaline, septate, branched fungal hyphae seen
3	17	Male	*Candida *spp.	No fungal element seen
4	32	Male	Aspergillus flavus	No fungal element seen
5	24	Male	*Syncephalastrum* spp.	No fungal element seen
6	24	Male	*Fusarium* spp.	Hyaline, septate, branched fungal hyphae seen
7	32	Female	Aspergillus niger	No fungal element seen
8	74	Female	*Candida *spp.	No fungal element seen

**Figure 1:**
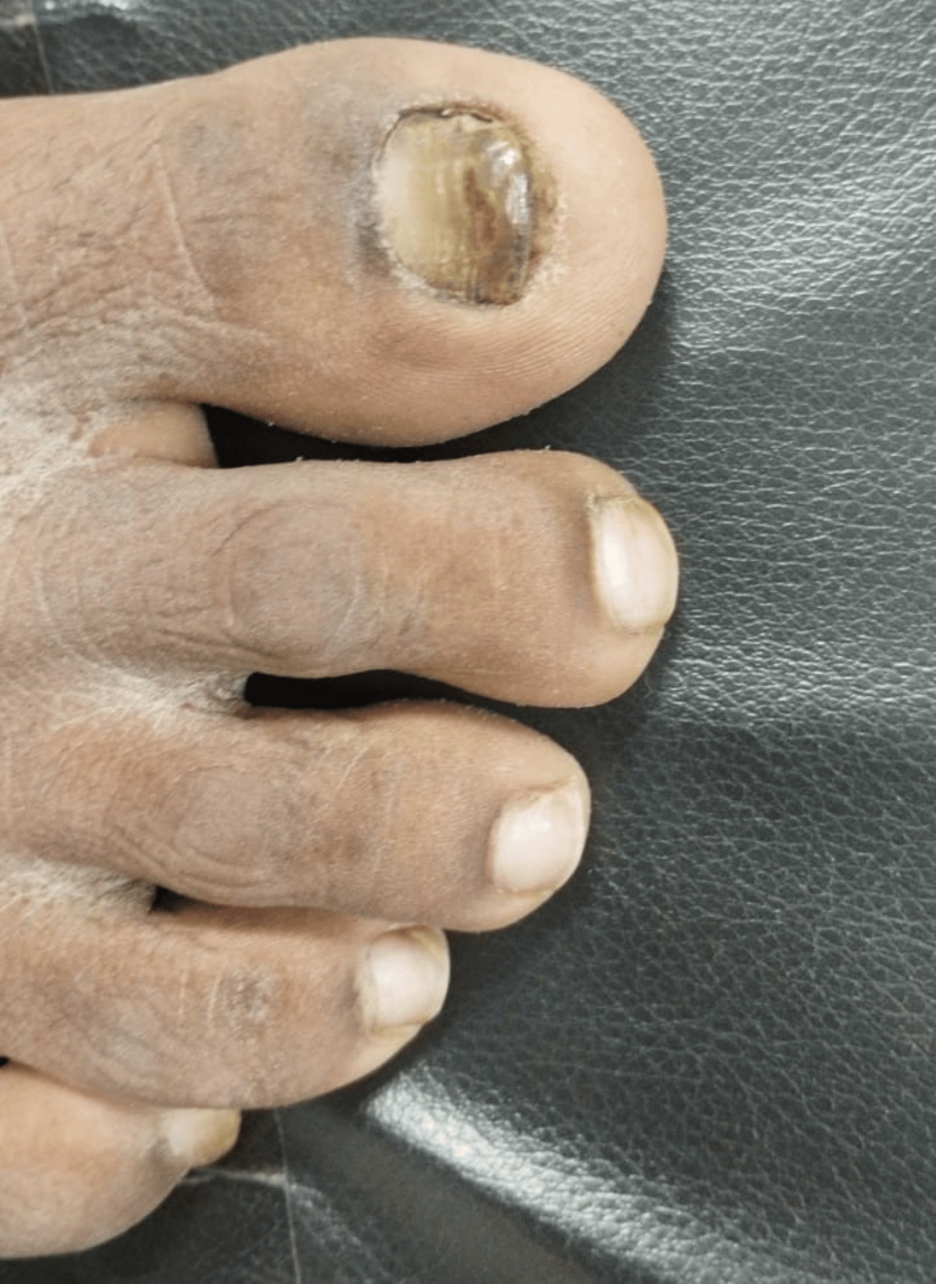
Discolored (brown chromonychia), right toenail of a 24-year-old male patient in which Syncephalastrum spp. grew on culture (nail specimen from patient #5, Table 1)

In three out of eight (37.5%) culture-positive specimens for non-dermatophytes, fungal elements were also detected by direct microscopy (KOH). In one additional nail specimen, hyaline, branched, septate hyphae were seen on direct microscopic examination (KOH), but no fungal growth was observed on culture. The fungus *Syncephalastrum* was detected in a 24-year-old adult male patient presenting with dull aching pain in the right toenail for the last six years (Figure 1). 

**Figure 2:**
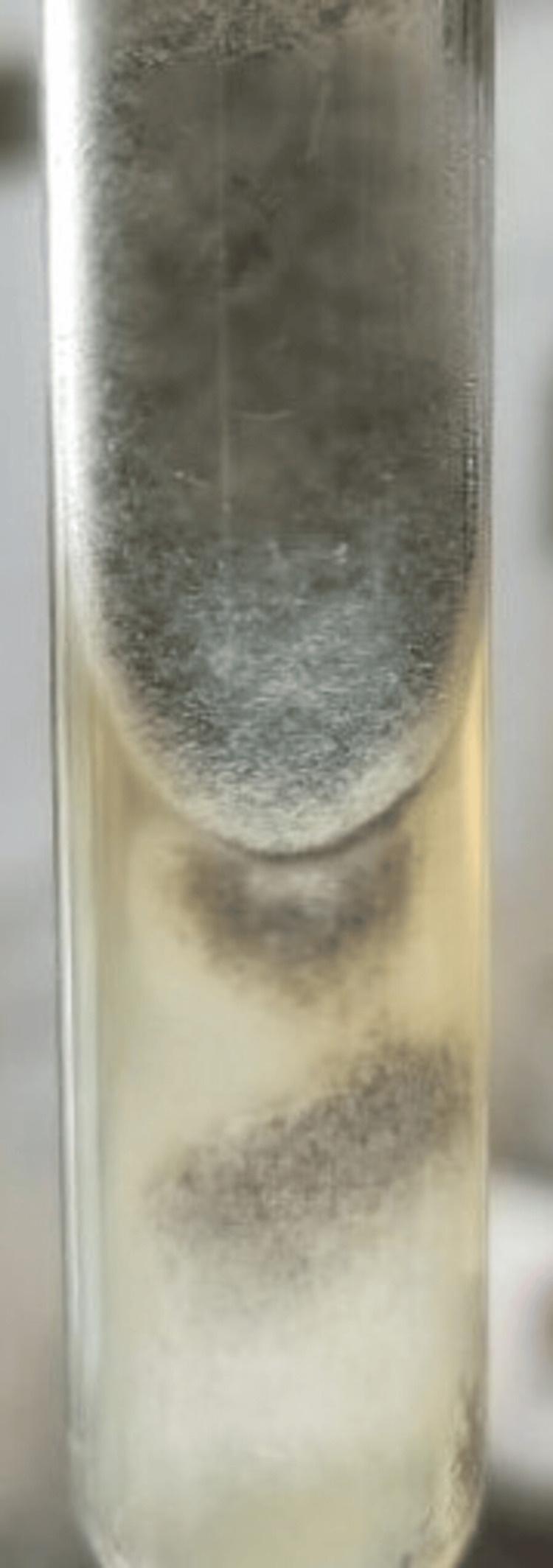
Culture growth on Sabouraud dextrose agar (nail specimen from patient #5, Table 1)

**Figure 3:**
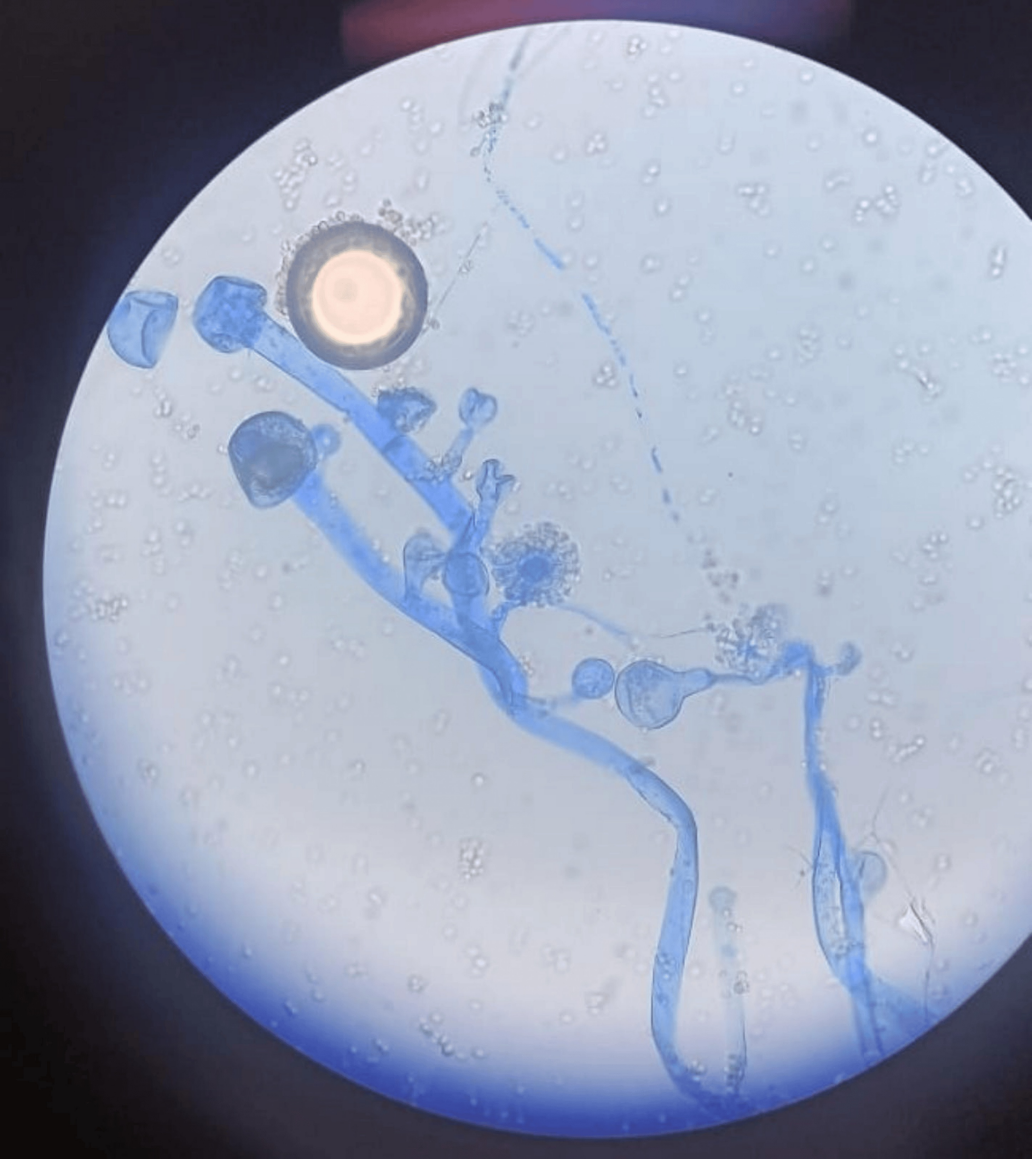
Lactophenol cotton blue mount preparation showing cylindrical merosporangia of Syncephalastrum spp. (nail specimen from patient #5, Table 1)

*Syncephalastrum* was grossly identified by cottony to fluffy colonies, white to light grey, and microscopically by the presence of cylindrical merosporangia (finger-like) on a terminal swelling of the sporangiophore (Figures 2-3).

Out of eight culture-positive specimens for non-dermatophyte fungi, five were males and three were females. The most common age group in cases of non-dermatophyte onychomycosis was found to be 20-40 years.

## Discussion

Initially thought to be caused by dermatophytes predominantly, now, non-dermatophyte molds are increasingly being reported to cause onychomycosis. The recurrence rate is quite high, and hence, maintenance of hygiene should be prioritized [6]. Traditionally, dermatophytes, especially *Trichophyton rubrum* and *Trichophyton mentagrophytes*, have been considered to be the most common cutaneous fungal pathogens, responsible for causing about 90% of nail infections [7,8]. But recently, clinical and epidemiological research has shown that non-dermatophyte molds and yeasts account for 20-30% of onychomycosis cases [9]. Gupta et al. reported in their study that older males are 2.1 times more prone to onychomycosis in comparison to females [6]. In our study, we also detected male predominance as compared to females in cases of non-dermatophyte onychomycosis, with the adult population being affected more as compared to older individuals. Trauma to the nail is an important predisposing factor for onychomycosis [10].

In a non-dermatophyte mold-related onychomycosis study done by Bombace et al., 57.2% of the specimens collected from onychomycosis patients tested positive for both direct microscopic examination and non-dermatophyte mold culture isolation in ≥2 subsequent inoculations. Conversely, in 42.8% of the examined specimens, non-dermatophyte molds grew after ≥3 subsequent inoculations, though these specimens were initially negative in direct microscopic examination [11]. However, in our study, only 37.5% of culture-positive specimens for non-dermatophytes were also KOH-positive, underscoring the importance of performing composite testing for accurate diagnosis. To diagnose onychomycosis accurately, it is essential to perform a clinical examination first, followed by a mycological examination. It has been found that *Scytalidium* species, which is currently considered a primary nail pathogen, produces infections that clinically mimic the features of dermatophytosis [12].

The fungus *Syncephalastrum* belongs to the class *Zygomycetes* and the order *Mucorales*. Unlike other *Zygomycetes* that typically cause central nervous system and pulmonary infections, *Syncephalastrum *species are more prone to causing onychomycosis and dermatomycoses [13]. Mohanty et al. from Odisha, India, reported total dystrophic onychomycosis due to *Syncephalastrum racemosum* in a 41-year-old female who was successfully treated with oral pulse itraconazole and neodymium-doped yttrium aluminum garnet (Nd:YAG) laser therapy [10]. Amatya et al. reported a case of mycetoma-like lesions caused by* Syncephalastrum* species [13]. Recently, a case of total dystrophic onychomycosis of fingernails due to *Syncephalastrum racemosum* was reported by Kumaran and Rudramurthy from Kerala, India [14]. Kamalam and Thambiah from Madras, India, described a notable case of an opportunistic cutaneous infection affecting the thumb in an adult male with diabetic ketosis, attributed to *Syncephalastrum* spp. The fungus (isolated from the skin) was observed to produce arteritis in the dermal vessels. However, the patient succumbed to diabetes mellitus without any concurrent systemic mycosis [15]. Mangaraj et al. reported a case of subcutaneous mucormycosis (solitary swelling in the anterior chest wall) due to *Syncephalastrum racemosum *in an adult diabetic male [16].

 We have briefly summarized the relevant literature related to* Syncephalastrum* fungus, highlighting its role as a causative agent of onychomycosis in Table 2 [10,14,17-19].

**Table 2:** Case reports of onychomycosis caused by Syncephalastrum spp. Nd:YAG: neodymium-doped yttrium aluminum garnet; H/O: history of

Authors of the study	Publication year	Place	Age/sex	Site/type of infection/etiology	Ascertainment of fungal infection	Management	Comorbidities	Outcome	Comments
Mohanty et al. [10]	2019	Odisha, India	41/F	Right great toenail: total dystrophic onychomycosis:* Syncephalastrum racemosum*	Microscopy, culture	Oral pulse itraconazole and 1064 nm Q-switched Nd:YAG laser	-	Completely improved	H/O employment in fisheries involving prolonged immersion of feet in water
Jindal et al. [18]	2016	Punjab, India	48/M	Nails of the great toes of both feet and of the second toenail of the right foot: toenail onychomycosis: *Syncephalastrum racemosum*	Microscopy, culture	Fluconazole 150 mg orally for 4 weeks	H/O bronchial asthma for which he was taking corticosteroids on and off	Not mentioned	H/O trauma to nail
Baby et al. [17]	2015	Kerala, India	50/F	Left great toe nail: onychomycosis: *Syncephalastrum racemosum*	Microscopy, culture	Surgical excision, debridement, topical nystatin	H/O diabetes	Successfully treated	H/O injury to nail
Kumaran and Rudramurthy [14]	2014	Kerala, India	24/M	Nail dystrophy in the hands: total dystrophic onychomycosis: *Syncephalastrum racemosum*	Microscopy, culture	Fluconazole 150 mg orally for 4 weeks	-	Not mentioned	The patient was working as a construction laborer
Pavlović and Bulajić [19]	2006	Belgrade, Serbia, and Montenegro	45/M	Right great toenail: onychomycosis: *Syncephalastrum racemosum*	Microscopy, culture	Surgical extirpation of the nail plate, followed by nystatin ointment applied b.i.d. to the exposed nailbed for 2 weeks	-	Successfully cured	H/O injury to nail

Microscopically, in KOH mount, ribbon-like, broad, aseptate hyphae are usually seen, as in the case of other *Zygomycetes* members, but in LPCB mount, *Syncephalastrum *closely resembles *Aspergillus* and hence can be misidentified due to the presence of radially arranged merosporangia surrounding the vesicle [17]. Therefore, it is essential to perform microscopic examination very carefully. Moreover, being a usual contaminant, a high index of suspicion, isolation in repeated specimen cultures, and clinical correlation are essential before the diagnosis is made [20].

Gupta et al. emphasized in their study that non-dermatophyte organisms are becoming increasingly prevalent as causative agents of onychomycosis [7]. The emergence of non-dermatophyte onychomycosis may be due to improved techniques for the detection of fungal infections [18]. Apart from nail avulsion and debridement, the most commonly used oral therapeutic options for onychomycosis are terbinafine, fluconazole, and itraconazole [21]. Some topical treatment options include efinaconazole, ciclopirox, tavaborole, and amorolfine. Terbinafine and itraconazole (systemic antifungals) are prescribed for severe onychomycosis, while ciclopirox 8%, efinaconazole 10%, and tavaborole 5% are topical treatment options for mild-to-moderate onychomycosis (US FDA-approved) [6,21]. Despite initial treatment success, many patients experience a return of the fungal infection, necessitating ongoing management and potentially repeated courses of therapy. Addressing the high rate of recurrence is crucial for achieving long-term resolution and improving patient outcomes in the treatment of onychomycosis.

The limitations of the present study are its retrospective nature and small sample size. In addition to being a single-center study, its findings may not reflect broader population trends. These limitations suggest that while the study provides valuable insights into non-dermatophyte onychomycosis at a specific institution, future research with larger, multicenter cohorts and comprehensive diagnostic approaches is recommended to enhance the understanding and management of this condition.

## Conclusions

Non-dermatophyte molds are emerging as potential etiological agents of onychomycosis with the advancements in diagnostic modalities and heightened awareness among dermatologists and microbiologists. While there are various treatment options available for onychomycosis, the recurrence of the infection remains a significant challenge.
